# Molecular phylogeny and phylogeography of genus *Pseudois* (Bovidae, Cetartiodactyla): New insights into the contrasting phylogeographic structure

**DOI:** 10.1002/ece3.3269

**Published:** 2017-07-31

**Authors:** Shuai Tan, Zhihong Wang, Lichun Jiang, Rui Peng, Tao Zhang, Quekun Peng, Fangdong Zou

**Affiliations:** ^1^ Sichuan Key Laboratory of Conservation Biology on Endangered Wildlife Key Laboratory of Bio‐resources and Eco‐environment (Ministry of Education) College of Life Sciences Sichuan University Chengdu China; ^2^ Department of Biomedical Science Chengdu Medical College Chengdu Sichuan China

**Keywords:** biogeography, blue sheep, genus *Pseudois*, mitochondrial DNA, phylogenetic relationship

## Abstract

Blue sheep, *Pseudois nayaur*, is endemic to the Tibetan Plateau and the surrounding mountains, which are the highest‐elevation areas in the world. Classical morphological taxonomy suggests that there are two subspecies in genus *Pseudois* (Bovidae, Artiodactyla), namely *Pseudois nayaur nayaur* and *Pseudois nayaur szechuanensis*. However, the validity and geographic characteristics of these subspecies have never been carefully discussed and analyzed. This may be partially because previous studies have mainly focused on the vague taxonomic status of *Pseudois schaeferi* (dwarf blue sheep). Thus, there is an urgent need to investigate the evolutionary relationship and taxonomy system of this genus. This study enriches a previous dataset by providing a large number of new samples, based on a total of 225 samples covering almost the entire distribution of blue sheep. Molecular data from cytochrome *b* and the mitochondrial control region sequences were used to reconstruct the phylogeny of this species. The phylogenetic inferences show that vicariance plays an important role in diversification within this genus. In terms of molecular dating results and biogeographic analyses, the striking biogeographic pattern coincides significantly with major geophysical events. Although the results raise doubt about the present recognized distribution range of blue sheep, they have corroborated the validity of the identified subspecies in genus *Pseudois*. Meanwhile, these results demonstrate that the two geographically distinct populations, the Helan Mountains and Pamir Plateau populations, have been significantly differentiated from the identified subspecies, a finding that challenges the conventional taxonomy of blue sheep.

## 1 INTRODUCTION

**Figure 1:**
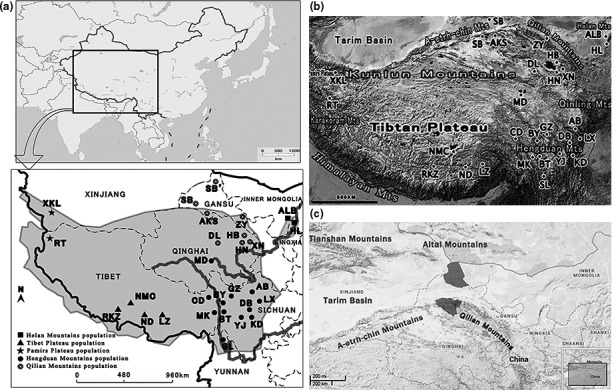
(a) Overview of the sampling localities for this study and the approximate geographic distributions of blue sheep (in gray). More specific locality data are shown in Table 1 and the different mitochondrial lineages have been labeled by different marker, respectively. (b) The topographic map of the distribution range of blue sheep. (c) The topographic map of Northwest China; the range of Subei County which is a special sampling site includes the separated two gray parts in the map

As a unique species in genus *Pseudois* popularly known as “blue sheep,” *Pseudois nayaur* is endemic to the Tibetan Plateau and the surrounding mountains (Mts.) (Figure 1a,b). The Tibetan Plateau is considered the highest ecosystem in the world and the largest high‐elevation ecosystem, with an average elevation of more than 4,000 m (Guo et al., 2006; Figure 1b). Unfortunately, due to the inaccessibility of the habitat of blue sheep, sample collection is particularly difficult, and related studies are limited. Classical morphological taxonomy has been utilized to suggest two identified subspecies in genus *Pseudois* (Bovidae, Artiodactyla), *Pseudois nayaur nayaur* and *Pseudois nayaur szechuanensis*. However, the validity of this taxonomy and the geographic characteristics of the two subspecies have never been carefully discussed and analyzed. Previous studies mainly focus on the vague taxonomic status of dwarf blue sheep, whose body size is notably smaller than that of common blue sheep (Cao, Wang, & Fang, 2002; Feng, Lajia, Taylor, & Webster, 2001; Liu et al., 2011). Intraspecific differentiation has rarely been addressed in previous studies due to the great difficulty in sample collection, so the composition of species and subspecies in genus *Pseudois* is continuous controversial. Different geographically restricted populations of blue sheep (*Pseudois nayaur*), a Central Asian ungulate with unclear variation in morphology and phylogeographic structure, have been widely debated. Moreover, the ungulate herbivores have strong cascading effects on the history and evolution of ecosystems (Pringle, Young, Rubenstein, & McCauley, 2007) and blue sheep is the most widely distributed of Central Asian ungulate. Consequently, there is an urgent need to investigate the evolutionary relationship and taxonomic status of this genus.

As for the currently established subspecies of blue sheep, *P*.* n*.* nayaur* is found throughout the Tibetan Plateau, Bhutan, Nepal, northern Pakistan, and northern India, as well as probably in Tajikistan (Harris, 2014; Figure 1a). Before 2012, the molecular and morphological data of this subspecies were always poorly documented. In our previous study, we showed that the subspecies *P*.* n*.* nayaur* was recognized in our molecular analyses for the first time. Nevertheless, only one sample of this subspecies was included in that study. Consequently, this result must be strengthened by accumulating more morphological and molecular data. The other subspecies, *P*.* n*.* szechuanensis* which is distributed in several provinces in western China (Figure 1a), has been the research interest of previous studies. In accordance with the previous morphometrics study (Yang, 2001), the skull of Ningxia (Helan Mts.) blue sheep is remarkably different from those of Sichuan, Gansu, and Qinghai sheep. The Helan Mts. population is considered to be the eastern peripheral population of *P*.* n*.* szechuanensis*. As shown in Figure 1a, this population has been completely geographically isolated from other populations. In 2001, an accumulation of data showed that the characters of the blue sheep population from Helan Mts. differed from those from other localities in behavior, ecology, and morphology (Wu, 2003; Yang, 2001), suggesting that this population never belonged to *P*.* n*.* szechuanensis*. Based on two mitochondrial (mt) genes, Zeng, Xu, Yue, Li, and Zou's (2008)finding subsequently indicated that the Helan Mts. population could likely be classified as a new subspecies according to the recognized 75% rule, which has been frequently used for the identification of subspecies (Amadon, 1949; Mayr, 1963). More samples should be accrued to investigate the phylogenetic relationship of this proposed new subspecies. So far, there has been little information about the molecular phylogeny and phylogeography of this species, and no new species and subspecies have been precisely designated. In this study, we collected a large cohort of samples (56) covering the entire distribution of blue sheep, specifically a large number (42) from the Helan Mts. population and three first‐time collected samples from Yunnan Province (Figure 1a). Along with the available sequences obtained from the previous studies, this study has three primary aims: The first is to discuss the genetic differentiation and diversity among the different populations of blue sheep, the second is to evaluate the level of genetic variation, species or subspecies within the geographically distinct population of blue sheep based on additional samples, and the third is to infer the phylogeography and diversification of geographically distinct populations in line with molecular dating data and historical biogeography results.

## 2 MATERIALS AND METHODS

### 2.1 Ethics statement

We only used wild blue sheep which died a natural death in this study. We collected these samples from the remains ourselves and it is confirmed that they had sacrificed in the field. All samples were obtained following the regulations for the implementation of China on the protection of terrestrial wild animals (State Council Decree [1992] No. 13). All our observational and field studies and laboratory work were approved by Wildlife Protection Office, Sichuan Provincial Forestry Departments (China), and by the Ethics Committee of Sichuan University, China.

### 2.2 Sample collection and DNA extraction

In this study, we expanded previous datasets by providing a large number (56) of new samples, including some areas (new six sampling locations) that had not been investigated previously (Table 1). We aimed to cover entire geographic regions across the range of blue sheep and to include samples in the vicinity of geographic breaks (Figure 1a). These samples were ascribed to different populations according to their geographic provenance (Figure 1a) and a comparative examination of specimens relative to original descriptions from previous phylogenetic studies. These samples belonging to 27 sampling locations were divided into five groups: the Helan Mts. (152 samples, two sampling locations), Hengduan Mts. (39 samples, 13 sampling locations), Tibetan Plateau (11 samples, four sampling locations), Qilian Mts. (15 samples, nine sampling locations), and Pamir Plateau populations (eight samples, two sampling locations). We discarded any unreliable samples that were not personally collected by our group, and the sample locations of 64 cytochrome *b* (Cyt *b*) and 32 mitochondrial control region (D‐loop) haplotype sequences (Table 1) are graphically indicated in Figure 1. Entire muscle samples were preserved in 95% ethanol at room temperature during transport and at −80°C in the laboratory. MtDNA was extracted from muscle and skull tissues according to the protocol detailed in the Genomic DNA Extraction Kit (Tiangen, China); skulls were first pulverized and soaked in EDTA for 72 hr.

**Table 1:** Geographic sites and sequence information of all samples included in this study

Sample group	Sample region	Sample number	Name of haplotypes	Sample locality	GenBank accession number	
					Cyt *b*	D‐loop
Helan Mts.	Ningxia	6	HL1~HL6^a^	Helan Mts.	AF493575~AF493580	
	Ningxia	29	HL11~HL18^b^	Helan Mts.	JQ406567~JQ406574	
	Ningxia	45	HL7~HL10^c^,^d^	Helan Mts.	EU571712/KJ467201~KJ467203	KX641001
	Ningxia	71	HL11~HL22	Helan Mts.		DQ234676~DQ234690
	Inner Mongolia	1	ALB^b^	Alxa Left Banner	EU571213	
Hengduan Mts.	Sichuan	9	BT1~BT7^d^	Batang County	EF420234~EF420235/JN839961~JN839965	EF420236~EF420237/JN8399986~JN8399989
	Sichuan	2	GZ^d^	Ganzi County	JN839982	JN840002
	Sichuan	4	CQ/LX3^d^	Chongqing Zoo	JN839966	JN840006~JN840009
	Sichuan	1	DB/BT3^d^	Danba County	JN839981	JN839995
	Sichuan	2	BY^d^	Baiyu County	JN839975	JN840000
	Sichuan	5	KD1~KD3^c^,^d^	Kangding County	JN839976~JN839977,KJ467208	JN839992~JN839993/KX641000
	Sichuan	3	LX1~LX3^d^	Lixian County	EU571711/JN839970~JN839971	EF420239/JN839991~JN839991
	Sichuan	1	YJ^d^	Yajiang County	EU571710	EF420238
	Sichuan	1	AB^b^	Ngawa County	JQ406566	
	Yunnan	3	SL^c^,^d^	Shangri‐la County	KJ467209	KX640999
	Tibet	4	CD1~CD3^c^,^d^	Changdu County	JN839984/KJ467206~KJ467207	JN840001/KX640998
	Qinhai	3	MD1~MD3^b^	Maduo County	JQ406556~JQ406558	
	Tibet	1	MK^d^	Mangkang County	JN839985	JN840005
Tibet Plateau	Tibet	3	ND1~ND3^c^,^d^	Naidong County	JN839983/KJ467201	JN840004/KX640995~KX640996
	Tibet	3	NMC^b^	Namtso	JQ406562	
	Tibet	2	LZ1~LZ2^b^	Nyingchi	JQ406564~JQ406565	
	Tibet	3	RKZ^b^	Shigatse	JQ406561	
Qilian Mts.	Qinhai	2	DL1~DL2^d^	Dulan County	JN839973	JN839994
	Qinhai	1	HN^d^	Hainan AP	JN839980	JN839997
	Qinhai	1	HB^d^	Haibei AP	JN839979	JN839999
	Qinhai	1	XN^d^	Xining Zoo	JN839979	JN839998
		1	TJ^d^	Tianjun County		JN839966
	Gansu	1	GS^d^	Gansu Province	JN839974	JN840003
	Gansu	3	SB1~SB2^b^	Subei County	JQ406551~JQ406552	
	Gansu	3	AKS1~AKS2^b^	Akese County	JQ406553~JQ406555	
	Gansu	2	ZY^c^,^d^	Zhanye	KJ467205	KX640997
Pamir Plateau	Xinjiang	6	XLK1~XKL2^b^	Western Kunlun Mts.	JQ406559~JQ406560	
	Tibet	2	RT^b^	Ritu County	JQ406563	

a Cao et al. (2004).

b Li et al. (2012).

c New samples in this study.

d Our laboratory's sample.

### 2.3 Amplification and sequencing

The entire Cyt *b* gene sequences were amplified using the primer pairs L14724 and H15915 (Zeng et al., 2008) and CYF and CYR (Tan et al., 2012), while the primers D‐LOOP_L2 and D‐LOOP_H2 were used to amplify the 5′ section of the mitochondrial control region (Wang, Cao, Liu, & Fang, 2006), which includes 554 bp of the hypervariable segment (Eythorsdottir & Tapio, 2001). Amplification was performed in a total volume of 50 μl containing 50 mm KCl, 10 mm Tris–HCl, 1.5 mm Mg^2+^, 200 μmol of each dNTP, 0.2 μmol of each primer, 1 U Taq DNA polymerase (TaKaRa, China), and approximately 10 ng of genomic DNA. Amplification was performed for 35 amplification cycles on a thermocycler with denaturation at 94°C for 30 s, annealing at optimal temperature for each primer pair for 45 s, and extension at 72°C for 2 m, with a final extension at 72°C for 7 m. Successful PCR products were purified and sequenced using the same PCR primers on an ABI PRISM 3730 (Applied Biosystems, Inc.).

### 2.4 Phylogenetic analyses

The obtained Cyt *b* and D‐loop sequences were blasted using the software MEGA v.6.06 (Tamura, Stecher, Peterson, Filipski, & Kumar, 2013) and aligned with the previous sequences from GenBank (Table 1) using the Clustal W algorithm (Higgins et al., 1994) with default parameters and no deletions, insertions, or stop codons present in this alignment. Haplotypes were detected using DnaSP v.5.10.01 software (Librado & Rozas, 2009), and duplicate sequences were not used for construction of phylogenetic trees. For the phylogenetic analyses, we chose maximum likelihood (ML) and Bayesian inference (BI) using the programs jModeltest software v.0.1.1 (Posada, 2008) to determine the appropriate model of molecular evolution in a likelihood ratio test framework based on the Akaike information criterion (Posada & Buckley, 2004). To generate robust trees, the sequences of goat (*Capra hircus*) and aoudad (*Ammotragus lervia*) were chosen as outgroups. ML analysis was performed with PhyML software v.3.0 (Guindon & Gascuel, 2003) allowing four substitution rate categories, and the final tree was computed from 1,000 bootstrap replicates. BI analysis was carried out using MrBayes software v.3.1.2 (Huelsenbeck & Ronquist, 2001), and the Markov chain Monte Carlo (MCMC) sampling approach was used to calculate the Bayesian posterior probabilities. The program was started with random trees and sampled every 100 generations for a total of 3,000,000 generations. Then, the first 25% of trees sampled were discarded as burn‐in. To ensure searching for the stationary parameter of Markov chain, Tracer software v.1.4 (Rambaut & Drummond, 2007) was employed to verify that sampled values of log likelihood plotted against generation time reached a stable equilibrium. Trees in this analysis that biased the equilibrium were not used to calculate the posterior probabilities. The retained trees were then used to generate a consensus tree in combination with the Bayesian posterior probabilities.

### 2.5 Sequence analyses

The nucleotide and haplotype diversity of Cyt *b* sequences were calculated using DnaSP software v.5.10.01 (Librado & Rozas, 2009). To assess the possibility of sudden population expansion, Tajima's *D* neutral test was implemented to detect different population expansions. For Tajima's *D*, 1,000 coalescent simulations were performed with 95% confidence intervals using DnaSP v.5.10.01. They were not calculated for the Pamir Plateau population because of the limited number of samples. These parameter values are summarized in Table 2. A calculation of average sequence divergence among different populations with maximum composite likelihood model and Kimura 2‐parameter (K2P) distances was performed using MEGA v.6.06 (Tamura et al., 2013; Table 3).

**Table 2:** Molecular diversity indices for Cyt b sequences of blue sheep

Population	*n*	*N*	Hd	p	*D*	Fu's Fs
Helan Mountains Population	81	19	0.811 (0.007)	0.00531 (0.00049)	−0.59731	−0.975
Hengduan Mountains Population	39	26	0.974 (0.013)	0.01978 (0.00078)	−0.43189	−1.461
Qilian Mountains Population	14	11	0.967 (0.037)	0.01528 (0.00187)	−0.97396	−1.293
Sichuan Population^a^	53	37	0.984 (0.008)	0.02152 (0.00067)	−0.88272	−4.714
Tibet Plateau Population	11	6	0.873 (0.071)	0.00955 (0.00094)	0.29337	2.930
Overall Population	145	62	0.981 (0.003)	0.02471 (0.00121)	−0.72100	−3.458

a Sichuan population include the Hengduan Mts. population and Qilian Mts. population.

Number of individuals (*n*), number of haplotypes (*N*), haplotype diversity (Hd), nucleotide diversity (p), Tajima' s *D* value (*D*), and Fu's Fs value (Fu's Fs) are shown (standard deviations are in parentheses).

**Table 3:** Genetic distances within and between different populations included in this study

	1	2	3	4	5	6
1—Tibet Plateau population	*0.010*	**0.038**	**0.044**	**0.048**	**0.041**	**0.084**
2—Helan Mts. population	0.038	*0.005*	**0.035**	**0.037**	**0.037**	**0.082**
3—Hengduan Mts. population	0.045	0.035	*0.021*	**0.025**	**0.042**	**0.088**
4—Qilian Mts. population	0.048	0.037	0.025	*0.019*	**0.043**	**0.093**
5—Pamir Plateau population	0.041	0.037	0.042	0.043	*0.002*	**0.088**
6—Outgroup goat	0.084	0.082	0.088	0.093	0.088	*NA*

Distances calculated using MEGA's maximum composite likelihood model with rate variation among sites defined by the gamma shape parameter are above the diagonal. Mean maximum‐likelihood divergences within each population are in the diagonal with bold text. K2P distances are below diagonal.

### 2.6 Molecular dating

**Figure 2:**
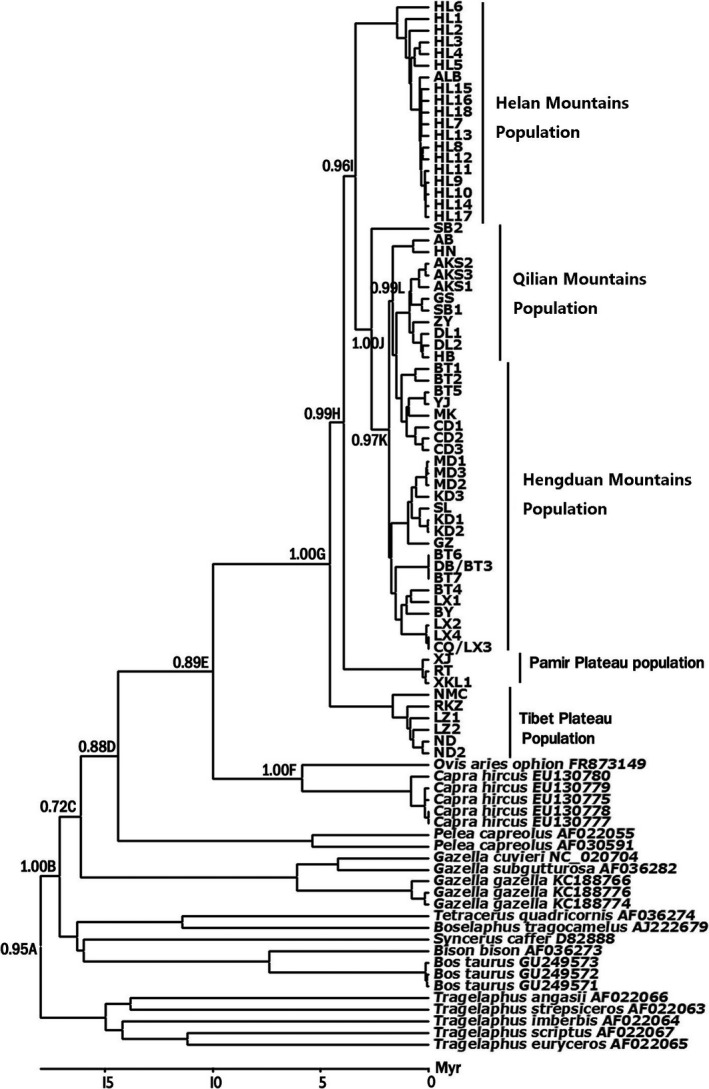
Divergence time estimates of family *Bovidae* and blue sheep based on Cyt *b* gene (Table 4). Numbers above branches refer to the posterior probabilities (PP) and letters are node names as in Table1

Divergence dates were estimated using a Bayesian analysis of Cyt *b* sequences implemented using BEAST software v1.7.5 (Drummond & Rambaut, 2007). The program BEAST is known as a unique program to simultaneously build phylogenetic trees and estimate divergence dates. At present, there have been few studies about the genus *Pseudois*, so we chose three fossil calibration points of other species in Bovidae for estimating the time of divergence. These three important fossil calibration points in analyses have been reported, and they were incorporated as upper and lower constraints: (i) The split between Bovinae and Antilopinae (16.4–23.8 Mya (millions of years ago)) was used by Hassanin and Douzery (2003) (Node A in Figure 2). (ii) The emergence of Bovinae tribes dates back to 11.5–19.7 Mya (Hill et al., 1985; also see Hassanin & Douzery, 2003; Node B in Figure 2). (iii) The calibration of 5–7 Mya for the sheep–goat split was derived from the fossil record (Carroll, 1988; Savage & Russell, 1983; Node F in Figure 2). In BEAST, a Yule process speciation prior and an uncorrelated lognormal model of rate variation were employed in each analysis. In the MCMC analysis, following a discarded burn‐in of 1,000,000 steps, the posterior samples were drawn at every 1,000 steps of a total of 10,000,000 steps. This analysis was repeated, and the samples from these two runs were combined. Convergence was assessed in Tracer v1.5 (Rambaut & Drummond, 2007), and the effective sample sizes of parameters sampled from MCMC in our run were greater than 200. The means between the upper and lower bounds of the 95% highest posterior density interval for divergence times were calculated using the program TRACER. FigTree v1.4.0 (Rambaut, 2012) and TreeAnnotator v1.7.5 (Drummond & Rambaut, 2007) were used to assess the tree topologies.

### 2.7 Biogeography

To infer the historical biogeography in this study, we performed a Bayesian binary MCMC (BBM) analysis implemented in Reconstruct Ancestral States in Phylogenetics v2.1 beta (RASP) to reconstruct geographic areas at nodes in the phylogeny (Yu, Harris, Blair, & He, 2015). This method calculates the frequencies of ancestral distributions, ranges, or other traits at each node and averages them over all sampled trees. We used the two Cyt *b* trees obtained from former phylogenetic analyses and geographic area codes. The BBM was run in parallel for 5 million generations with 10 chains under the Jukes–Cantor fixed‐state frequencies model and among‐site variation (Gamma) set to equal.

## 3 RESULTS

### 3.1 Phylogenetic analyses

**Figure 3:**
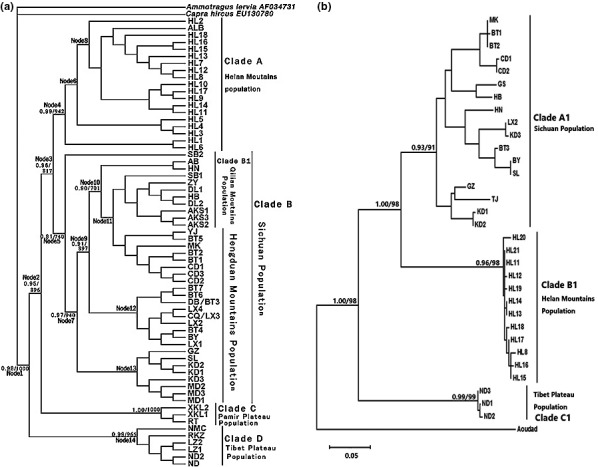
(a) Phylogenetic trees based on the Cyt *b* sequences for the genus *Pseudois*. The same topology retrieved by the Bayesian inference and maximum likelihood. Values above or below the branches represent Bayesian posterior probabilities (PP = 1) and maximum‐likelihood bootstrap values (BP = 1,000). Inferred ancestral distribution at each node of the blue sheep phylogeny estimated by BBM analysis implemented in RASP. The results are shown in Table 5. (b) Phylogenetic tree reconstructed using D‐loop sequences from blue sheep and dwarf blue sheep. The posterior probabilities and bootstrap support values are shown on the nodes

Combined with other corresponding available sequences from GenBank, haplotypes from 64 Cyt *b* sequences and 32 D‐loop sequences were obtained for phylogenetic tree reconstructions. Identical patterns and topologies were collected, with strongly supported bootstrap values and posterior probabilities. Both Cyt *b* trees (Figure 3a,b) showed deep genetic structure across the range of the blue sheep complex. As shown in Figure 1a and Table 1, four major monophyletic haplogroups were revealed, and they were designated as clades A, B, C, and D. The lineage in Helan Mts. population (Clade A) was consistently recovered with a large number of new samples. Similarly, the earliest‐diverging clade was confined to the Tibetan Plateau population (Clade D), which was recognized as the sister group to the rest of the complex. Another differentiated population (Clade C) was found to be mostly restricted to the Pamir Plateau, that is, the gap between the Karakorum Mts. and the West Kunlun Mts. that probably separates Clade C from other populations. The last was an aggregate of all individuals from the Qilian Mts. and the Hengduan Mts. (Clade B), including seven entire samples of dwarf blue sheep (BT1–BT7). Therefore, the two originally designated subspecies of blue sheep, *Pseudois nayaur nayaur* and *Pseudois nayaur szechuanensis*, as well as the new subspecies in this genus, were established in the phylogenetic trees. Except for AB and SB2, Clade B could be further divided into four subclades, namely Clade B1 (the group in the Qilian Mts.) as well as clades B2, B3, and B4 (without geographic specificity). All of these clades belonged to the Hengduan Mts. population. Among blue sheep, haplotypes sampled from all populations formed deeply divergent and strongly supported clades, with two exceptions (Table 1; Figure 1a). The first exception involved a haplotype from Ngawa County (AB), with its sampling point located in the Hengduan Mts. but clustered with the individuals forming the Qilian Mts. population. The other possible exception involved one samples from Subei County (SB2) that formed a sister group to the lineage of other individuals from the Qilian Mts. and the Hengduan Mts. The D‐loop trees illustrated the three major clades, which were designated as clades A1, B1, and C1 (Figure 3b) because we did not obtain the D‐loop sequence from the Pamir Plateau. The three monophyletic haplogroups also belonged to three geographic populations, Clade A1 (Helan Mts.), Clade B1 (Sichuan population), and Clade C1 (Tibetan Plateau).

### 3.2 Genetic diversity and mtDNA sequence characteristics

In addition to the marked phylogeographic structure described above, all the populations exhibited relatively high levels of haplotype and nucleotide diversity (Table 2). Among the 153 sampled blue sheep, we found 62 Cyt *b* haplotypes, with none shared by major clades. Nucleotide diversity was similar among these populations (Table 2) but was slightly higher for the Sichuan population (Clade B). Analyses of haplotype and nucleotide diversity as well as Tajima's *D* neutral test and Fu's Fs test were not conducted for Clade C because of the limited number of samples. According to Tajima's *D*‐test and Fu's Fs test, *p*‐values for all the three clades (clades A, B, and C) were not significant (*p* > .1). Except for the Tibetan Plateau population (Clade D), the significantly negative Fs and Tajima's *D* suggested an expansion episode for the Helan Mts. and Sichuan populations, which simultaneously excluded the effect of background selection (Fu, 1997; Hull & Girman, 2005). We also observed relatively high levels of sequence divergence among the four previously established populations in the genus *Pseudois* (Table 3). The Hengduan Mts. and Qilian Mts. populations were attributed to the Sichuan population, leading to a low divergence between them.

### 3.3 Molecular dating and historical biogeography

Table 4 shows the posterior distribution results of the Bayesian inference of divergence times, which were also applied to obtain age estimates and 95% credibility intervals. The results indicated the divergence times among the different populations in the genus *Pseudois*. The Tibetan Plateau population was the first to split, at approximately 4.62 (2.51–6.73) Mya, followed by the Sichuan population at approximately 2.64 (1.58–3.69) Mya. The geographically distinct populations, the Helan Mts. and Pamir Plateau populations, were inferred to be separated at approximately 3.41 (2.14–4.68) Mya and 3.81 (2.26–5.36) Mya, respectively. It was estimated that the Qilian Mts. population was the last to isolate, at 1.68 (0.84–2.52) Mya. The results (see Table 5) of biogeographic analysis based on Bayesian Binary MCMC showed only the most likely states at each node in Figure 3b, which were used in the subsequent discussion.

**Table 4:** Molecular dating of the main splitting events within Bovinae and blue sheep

Node	Mean (Mya)	95% highest posterior density		Node	Mean (Mya)	95% highest posterior density	
		Lower (Mya)	Upper (Mya)			Lower (Mya)	Upper (Mya)
A	18.00	17.80	18.20	B	17.18	16.25	18.11
C	16.14	14.80	17.47	D	14.42	11.89	16.95
E	10.01	7.21	12.80	F	5.83	4.22	7.43
G	4.62	2.51	6.73	H	3.81	2.26	5.36
I	3.41	2.14	4.68	J	2.64	1.58	3.69
K	1.79	0.87	2.70	L	1.68	0.84	2.52

Mean, lower, and upper 95% highest posterior density values obtained in BEAST are shown. Dates are in millions of years (Mya) before present. Nodes are shown in Figure 3.

**Table 5:** Biogeographic analysis using Bayesian binary MCMC, showing only the most likely states at each node

Node	Distribution	Percentage	Node	Distribution	Percentage
1	D	0.68	2	D	0.80
3	E	0.66	4	A	0.60
5	C	0.74	6	A	1.00
7	B	0.79	8	A	1.00
9	B	0.99	10	B	0.68
11	B	0.74	12	B	1.00
13	B	1.00	14	D	1.00

Geographic area codes are as follows: A = Helan Mts., B = Hengduan Mts., C = Qilian Mts., D = Tibet Plateau, E = Pamir Plateau, F = Maduo County (three samples named MD in Figure 1a). Nodes are shown in Figure 2a.

## 4 DISCUSSION

Only two mitochondrial genes were previously used to study the molecular phylogeny of the genus *Pseudois*: Cyt *b* gene and mitochondrial control gene (Li, Liu, Wang, & Huang, 2012; Tan et al., 2012; Zeng et al., 2008). Based on the expanded taxonomic and geographic sampling, our ML and BI analyses of the Cyt *b* gene provided a more robust signal than all previously identified relationships (clades A, B, D, Helan Mts. Population, Tibetan Plateau population, and Hengduan Mts. Population) and revealed the existence of a new large clade, that is, the Pamir Plateau population (Clade C). These four distinct monophyletic lineages were restricted to different geographic regions (as shown in Figure 3a). The sampling locations of D‐loop sequences were limited, and there were no D‐loop sequences from the Pamir Plateau sample, but the D‐loop phylogenetic trees also suggest that the three monophyletic groups belonged to the three geographic populations (Figure 3b). The two identified subspecies, *Pseudois nayaur szechuanensis* and *Pseudois nayaur nayaur*, were classified as Clade B and Clade D, while the two putative new geographically distinct populations, the Helan Mts. and Pamir Plateau populations, were designated as Clade A and Clade C, which were, respectively, located at the east and west ends of the blue sheep distribution area (Figure 1a). This phenomenon strongly supported the concept that genetic differentiation and isolation frequently occurred among the different geographic populations of this widely distributed species, especially for the peripheral populations (García‐Ramos & Kirkpatrick, 1997). Genetic distance is usually used as a standard for species distinctiveness based on the already recognized concepts of major species, such as the evolutionary and biological concepts of species (Mayr, 1963; Wiley, 1978). The main criterion of some phylogenetic species concepts was identified as the evidence of genetic isolation among different monophyletic clades (Mishler & Theriot, 2000). The results of the comparisons of the genetic distances among different monophyletic clades were in congruence with the Cyt *b* phylogenetic tree topology. The mean sequence divergence among monophyletic clades in the Cyt *b* phylogenetic tree was 3%–4% (Table 3), while the results were much lower than the mean value (6.3%–12.6%) of interspecific divergence in Bovidae (Schreiber, Seibold, Nötzold, & Wink, 1999). Meanwhile, these means fell in the range of Cyt *b* divergence values among subspecies in other related species of Artiodactyla, *Capra ibex* (4.258%–7.814%), *Moschus berezovskii* (2.18%), and *Sylvicapra grimmia* (2.8%) (Li et al., 2012). Thus, these results strengthened the reliability of our identified subspecies in phylogenetic trees, especially for the two suspected putative subspecies. As for the identified subspecies in genus *Pseudois*,* P*.* n*.* nayaur* was the earliest‐diverging lineage, which corresponds to the inference that blue sheep originated from the Tibetan Plateau and then dispersed northeastwardly (Schaller, 1998). Tibetan Plateau lies between the Himalayan Mts. to the south and the western Kunlun Mts. to the north (Figure 1b), with an average altitude of approximately 4,500–5,500 m. The plateau surface is complete with many interior river systems, lakes, and a wide meadow, forming an alpine tundra environment and a unique terrain that reduces species diversity. Based on limited data, the haplotype diversity of this population is much lower than the others. The significantly positive Fu's Fs and Tajima's D suggested that the Tibet population underwent balancing selection differently from the other populations (Table 2). As this population lived at such high elevations, they must have experience certain extreme environmental conditions, including hypoxia, high levels of UV radiation, and dramatic daily changes in temperature (Sun et al., 2015). In the present study, we collected wool samples from the Tibetan Plateau and Hengduan Mts. populations. The wool of Tibet blue sheep was denser and deeper in color than that of the Hengduan Mts. sheep. This morphological result also agreed with our molecular phylogenetic analysis regarding the status of the Tibetan Plateau population as a possible species or subspecies.

For the other identified subspecies *P*.* s*.* nayaur*, as mentioned above, Clade B contained the individuals from MD (Maduo County), the Qilian Mts. and the Hengduan Mts (as shown in Figure 3a). The individuals of the Qilian Mts. population formed a subclade with a haplotype (AB) from the Hengduan Mts. population, thus showing a far‐off distance (Figure 1a). In fact, the mountains in this region are continuous, without possible geographic barriers to prevent gene flow among these blue sheep of different populations in this area (Figure 1b). Owing to sampling locations, previous studies made no exact conclusions regarding the complex genetic structure of this recognized subspecies (*Pseudois nayaur szechuanensis*), but in the previous morphometrics study, the skull of Ningxia (Helan Mts.) blue sheep is remarkably different from those of Sichuan, Gansu, and Qinghai sheep. The same is our molecular data showing that most subclades are restricted to the Qilian Mts., with a slight genetic distance (2%) from the Hengduan Mts. population. Figure 1a,b clearly shows that the Qilian Mts. are located in Qinghai and Gansu provinces and that most of the Hengduan Mts. region are in Sichuan Province. Within the genus *Pseudois*, a blue sheep from the Himalayas (Bunch, Nadler, & Simmons, 1978), one from Qinghai Province (Li, 1999), and a dwarf blue sheep from Sichuan Province (Bunch, Wang, Zhang, Liu, & Lin, 2000) have been proven to share the same chromosome number of 2*n* = 54, whereas a blue sheep from Gansu Province has been uniquely found to have a different chromosome number, 2*n* = 56 (Bunch et al., 2000). These results support our molecular phylogenetic analysis, which showed that there is a significant genetic divergence between the two different populations of the Sichuan subspecies. One possible explanation for these results is the difference in the environments of their habitats. The Hengduan Mts. region is topographically complex: The elevation of the valley extends higher than 1,000−2,500 m, and the weather is typical of a monsoon climate. The Qilian Mts. from the northern outlier of the Kunlun Mts., with an average altitude of 4,000–5,000 m and a vegetation distribution showing an evident vertical gradient in a continental climate. Located in the Hengduan Mts., AB (Aba County) unexpectedly clusters with the individuals that form the Qilian Mts. population. The Sichuan population is composed of a recently suspected divergent subspecies, for which the molecular markers used were either too few or too conservative to determine the last coalescences or those still in the process of separation. Even so, more and further studies are required to define the broad distributional pattern of Clade B based on additional samples.

As for the two geographically distinct population, with additional Helan Mts. sheep samples (42), it was found that the Helan Mts. lineage (Clade A) had an arrangement congruent with recently published molecular phylogenies of blue sheep that constituted the immediate sister group of another population (Tan et al., 2012). Although the Helan Mts. population has been isolated from other populations and is located within the easternmost edge of blue sheep distribution (Figure 1a), it was recognized that this population belonged to the subspecies *P*.* s*.* nayaur* when it was discovered. Nonetheless, the molecular analyses showed that this isolated population had diverged significantly from other populations, while the inferred historical biogeography results demonstrated that it had migrated to the Hengduan Mts. and Qilian Mts. (Table 5). The molecular dating results indicated that the separation time between the Helan and Sichuan populations was approximately 2.64 (1.58–3.69) million years (Table 4). Through the long‐term gene flow and ecologically adaptive behavioral changes, this isolated population has developed a dramatic divergence from the other populations. Moreover, the data accumulated from these individuals presented differences in behavior, ecology, and morphology (Wu, 2003; Yang, 2001). Our results in the present study support the previous suggestions that the Helan Mts. population may have become a new subspecies or species of *Pseudois nayaur*.

As the world's highest plateau, the Pamir Plateau is located at the confluence of several of the world's highest mountain ranges, namely the Himalayas, Karakoram, and Hindu Kush. The average altitude of the region is greater than 5,000 m. Since Victorian times, this area has been known as the “Roof of the World”. The association of the Pamir Plateau with all three mountain ranges has generated a relatively high biodiversity. Compared with the Tibetan Plateau that lies to the east, Pamir Plateau's prominent vertical relief significantly increases the diversity of its natural habitat. However, as the altitude and climate of this area are too extreme for sampling, little molecular information on blue sheep has been reported to date. Furthermore, this plateau is a flat plateau (or U‐shaped valley) surrounded by mountains. Climate research has found that the Pamir region shows a strong phytogeographic affinity to the adjoining Tibetan Plateau, with a strong Mediterranean influence as well. The huge differences existing among the Pamir Plateau, the Tibetan Plateau, and the Karakoram Mts. probably contribute to the geographic isolation. In Caprinae, *Ovis ammon* (Argali sheep) not only has a distribution similar to that of blue sheep but also maintains a close relationship with it. The Pamir Plateau population of Argali sheep is classified as one subspecies related to geographically close populations such as the Tibetan Plateau and Tian Shan subspecies. Hence, we speculated that the population of Pamir Plateau blue sheep probably represents a new species or subspecies of *Pseudois nayaur*.

Blue sheep originated from the Tibetan Plateau and then dispersed northeastwardly (Schaller, 1998). In accordance with our phylogenetic results, the Tibetan Plateau population was the earliest‐diverging lineage. This split may have resulted from the uplift of this range, because the divergence between the Tibetan Plateau population and other populations dates back to 2.51–6.73 Mya (Table 4), corresponding to the conjectured beginning of the Pliocene uplift of the Tibetan Plateau (3.5–4.5 Mya; Zeng et al., 2000). As illustrated in Figure 1b, the south edge of the Tibetan Plateau was constituted by the Himalaya Mts., which formed an insuperable obstacle for blue sheep. For this reason, we speculated that this species migrated to the peripheral mountains of Tibetan Plateau, which have been reported to be zones of high seismic activity. Later, some geographically isolated populations, such as the Pamir Plateau and Helan Mts. populations, gradually formed a new subspecies.

One concern with regard to this species is its distribution range. A recent survey on large mammals in Central Asia discovered the existence of a blue sheep population in the Altai Mts., outside the recognized distribution of this genus. In our present study, an exception involving the Subei County (SB2) haplotype formed a group sister to the lineage of other individuals from the Qilian Mts. and the Hengduan Mts., while a haplotype from the same location (SB1) clustered with the individuals from the Qilian Mts. population. The K2P distance between SB1 and SB2 reached 2.2% (Table 3), which was a relatively high value for two haplotypes from the same location. This result was probably an artifact of our small set of genes available for these populations. Subei County is an interesting place composed of two separate areas (Figure 1c) with major differences in terrain and environment. Figure 1c shows that the southern part of Subei County was located in the northern Qilian Mts., while the northern part lies in the eastern Altai Mts. In this context, we were able to deduce that these two haplotypes might be collected from different geographic locations. Regarding the K2P distance for SB2 compared with the other groups, it was 2.1% for the Qilian Mts. population, 2.0% for the Helan Mts. population, 2.2% for the Sichuan Mts. population, and 2.9% for the Pamir Plateau population. Except for the 3.9% found in the Tibetan Plateau, the others were approximately equivalent. In this case, one could speculate that the new population probably lives in the Altai Mts. Combining these data with some survey information on the Altai Mts, Gu and Luo presented convincing evidence for a certain number of blue sheep populations in this area (Gu & Gao, 1989; Luo & Gu, 1991). We would like to raise doubt about the recognized distribution range of blue sheep and classification of the genus *Pseudois*. Because these areas are famous as “no man's land” and because it is difficult to obtain samples there, these populations of blue sheep are still largely unknown. Consequently, we highly recommend that more attention should be paid to these distant regions to maintain genetic diversity and protect this rare species.

## CONFLICT OF INTEREST

None declared.
